# THE ASSOCIATIONS OF HANDGRIP STRENGTH AND ANTHROPOMETRIC MEASUREMENTS WITH UPPER LIMB BODY FAT MASS IN OLDER ADULTS

**DOI:** 10.1093/geroni/igad104.3630

**Published:** 2023-12-21

**Authors:** Jethro Raphael Suarez, Joon-Hyuk Park, Renoa Choudhury, Jeffrey Stout, Chitra Banarjee, Ladda Thiamwong

**Affiliations:** University of Central Florida, Orlando, Florida, United States; University of Central Florida, Orlando, Florida, United States; University of Central Florida, Orlando, Florida, United States; University of Central Florida, Orlando, Florida, United States; University of Central Florida, Orlando, Florida, United States; University of Central Florida, Orlando, Florida, United States

## Abstract

Body Fat Mass (BFM) is a crucial body composition measurement serving as a predictor for various health conditions and mortality in older adults. However, limited research has been conducted to investigate how upper limb BFM associates with anthropometric measurements and handgrip strength (HGS) in older adults. In this cross-sectional study, 119 older adults, ranging from 60 to 96 years of age (93 women, 26 men; mean age 74.71 ± 7.35 years), were assessed. Body composition was evaluated using the portable Bioelectrical Impedance Analysis (BIA) device, InBody S10, and average HGS was measured on each side using a hand grip dynamometer (JAMAR 5030J1). Multiple linear regression analysis was performed and found a significant, positive association for age (β = 0.048 , p = 0.024), height (β = 0.649, p = 0.024), and BMI (β = 1.371, p < 0.001) with left arm BFM, while weight and left HGS showed a negative association (β = -0.152, p = 0.007; β = -0.070 , p = 0.004, respectively). Similar trends were seen for the right arm. However, height and right HGS became statistically insignificant (β = 0.536, p = 0.069; β = -0.046 , p = 0.054, respectively) for right arm BFM only. These results show that the independent variables may have the ability to predict upper limb BFM in older adults without the need for body composition measurement methods (e.g., BIA). Further research is warranted to examine the true predictive capacity of the independent variables in relation to upper limb BFM.

